# What is the accordion effect?: harmonizing Bratman’s principles F and D

**DOI:** 10.1186/2193-1801-2-279

**Published:** 2013-06-26

**Authors:** Sven Fockner

**Affiliations:** IKU Institut, Senefelderstr. 15, 73760 Ostfildern, Germany

**Keywords:** Joel Feinberg, Donald Davidson, Michael Bratman, Accordion effect, Responsibility

## Abstract

In an article about the accordion effect Michael Bratman pointed out some difficulties in Joel Feinberg’s original description of it, which he calls “language relativity”. In this comment I do not contend any of Bratman’s conclusions, but try to resolve the tension noted by Bratman. I believe that his analysis of Feinberg and Davidson is detrimental to the study of the accordion effect since the difficulties he mentions can be explained by the context in which Feinberg gives his account of the accordion effect. Because of the focus of his essay, Feinberg is using the accordion effect not, as most scholars after him have done, to redescribe actions to include their consequences, but to disassemble action sentences to find forms of causality “hidden” in them. Such a reading of Feinberg explains the odd features of his description, aligns it with later work on the topic and enables a uniform understanding of the phenomenon.

In 1949 H.L.A. Hart published a paper with the title “The Ascription of Responsibility and Rights” in which he stressed that action-sentences have the primary function of ascribing responsibility. It was in response to Hart’s thesis that Feinberg, in passing, gave a name to a well-known phenomenon, which is, since then, referred to as the accordion effect^a^. The basic idea is that one can ascribe to an agent the consequences caused by his actions. If Peter opens a door and startles Paul who suffers a heart attack and dies, “we can say that Peter’s opening the door caused his death, or that Peter’s startling him caused his death, or simply that Peter killed him (by doing those things).” (Feinberg 1970, 134). The varying volume of those action sentences is alluded to by the changing size of the accordion. Numerous scholars have discussed, critiqued or employed Feinberg’s “invention,” most notably Donald Davidson (1980).

There remained, however, substantial differences between Feinberg’s original conception and the one of subsequent authors, which were addressed by Michael Bratman (2006) in a penetrating article. He pointed out that for Feinberg, the accordion effect requires a specific causal verb to do the ascription (e.g. kill for causing death). If such a verb does not exist the accordion effect does not work. Feinberg does not give an example, but John Atwell in critiquing Feinberg mentions that running a stop light and thereby causing an accident cannot be substituted by „accidenting“ (Atwell, 337 n2). But even if a fitting transitive verb exists, there are cases where it cannot be substituted without distorting the meaning of the sentence. This typically happens in cases of interpersonal causation: causing someone to move his finger is not the same as moving someone’s finger. This leads to a rather complex rule, which Bratman calls *principle F*:

“When an agent acts and his act causes some change, X, in something, Y, there is frequently (though not always) a specific transitive causal verb, C_X_, associated with causing that upshot in Y, such that it is true that the agent C_X_ –ed Y. When there is such a causal verb such that it is true that A C_X_ –ed Y, we have an instance of the accordion effect”. (Bratman, 9).

The question, why the ascription of causal agency cannot happen through the generic verb “to cause” is not satisfactorily answered by Feinberg. Why is it a case of the accordion effect to say that Peter killed Paul, but not that Peter caused Paul’s death? Bratman finds the “language relativity” in Feinberg’s conception “artificial” and “idiosyncratic” (Bratman, 10–11) especially in comparison with Davidson’s version of the accordion effect (Bratman’s *principle D*), which simply holds that “an agent causes what his actions cause,” (Davidson, 53). It is possible, however, to read Feinberg in a way that will make sense of the exceptions to the accordion effect noted by Bratman as well as of one other, which is also introduced by Feinberg and raises similar questions.

We have already mentioned the first difficulty, namely that it is not clear, why the ascription of causal consequences of an action to the agent requires a specific causal verb to be existent and applicable. In addition, according to Feinberg, the accordion effect does not work with simple actions. The reason he gives is that simple actions have no causal components therefore “one cannot play the accordion with them” (Feinberg, 136). Obviously Feinberg, like Davidson, thinks that simple actions (raising one’s arm) cannot be squeezed down to e.g. he contracted his muscles thereby causing his arm to be raised. So the accordion effect cannot be used to narrow simple actions. This does not explain, however, why it should be impossible to stretch out the accordion i.e. to incorporate the consequences of a simple action into the action^b^.

What is one to make of this? Did the scholar, who christened the accordion effect, have an inherently flawed or at least crude conception of it? This would be a rather easy way out, but it doesn’t seem to be the right one. Since the problems in Feinberg’s description are not caused by excessive brevity or a disregard for details—they are not slip-ups—but are actively and consciously introduced, there must be some motive, some thought, some meaning behind them. What was Feinberg’s perspective on things that made it reasonable for him to insert these exceptions? In the remainder of the article I will try to present a reconstruction of the point of view from which Feinberg was discussing the accordion effect. This perspective can, I suggest, explain the exceptions which so far seemed idiosyncratical. My proposition is that *principle F* is nothing else but a reversal of *principle D*.

What is the purpose of Feinberg’s essay and what is his reason for the introduction of the accordion effect? The goal of Feinberg is to reinterpret Hart’s argument in a way that will avoid the criticism that was launched against it, namely that Hart’s thesis only works in case of offenses or sub-par performance. After an in-depth discussion of faults and offenses, which were the focus of Hart’s article, Feinberg in the second part of his paper is trying to establish a broader basis for ascriptiveness which includes non-faulty actions sentences. He is suggesting five different ways in which responsibility can be ascribed. The first and most simple option is the ascription of causality. To say that X (e.g. a low pressure system) is responsible for Y (e.g. the bad weather) basically means X caused Y. Causality, according to Feinberg, is “perfectly equivalent” (Feinberg, 132) to responsibility. While it is commonly agreed, that A caused X can ascribe responsibility, things are not so clear concerning the relation of agency and responsibility. Why should A did X ascribe responsibility in the same sense as A caused X does? Feinberg tries to solve this problem by a concept he calls causal agency, his second way of ascribing responsibility. Here the accordion effect with its problematic exceptions comes into play.

This context is necessary as a background to understand Feinberg’s usage of the accordion effect. Usually the accordion effect is understood as a tool, to redescribe actions in terms of their consequences. A lot of discussion about the legitimacy of such redescriptions followed the initial publication of Feinberg’s paper^c^. I call this the constructive or forward usage of the effect. It must be said, that in his wording and his examples, Feinberg mentions that the accordion can be used in this way. Yet what Feinberg wanted to achieve was not to create redescriptions of actions, but to show that ascriptions of agency are ascriptions of responsibility. The name of his second way already suggests, what will do the trick: causal agency. Feinberg is trying to show that in many cases causality is hidden in agency. “Ascriptions of causal responsibility, then, are often precisely equivalent to ascriptions of the second type, which I have called ascriptions of causal agency” (Feinberg, 134). He goes on to explain that causal responsibility and causal agency “both say something about causation, the one quite explicitly, the other in the language of agency or authorship.” According to Feinberg, A did X is often just an implicit form of saying A caused X which is agreed to be ascriptive.

Feinberg is introducing the accordion effect in the discussion of the second way of ascribing responsibility in order to show that attributions of agency are nothing else than attributions of causality, which is a form of responsibility. The logical conclusion is that to attribute causal agency is also a possible way of attributing responsibility, which was what Feinberg was looking for: (non-faulty) actions sentences that are ascriptive in Hart’s sense. His purpose causes Feinberg to approach the accordion effect from another direction than most of the scholars after him have. He is not using the accordion effect to construct action sentences that include causal consequences; he is using it to deconstruct action sentences in order to lay bare the causal connection hidden in them. I call this the deconstructive or backward usage of the accordion. This backward approach is motivated by his question: how do action sentences ascribe responsibility? The first answer is causality. Yet there are sentences which do not include causality on the surface, but which have causality hidden in them. By dissecting these sentences via the accordion effect, one can see that they also ascribe causality and therefore responsibility. This feature is overlooked in much of the discussion about Feinberg and causes the incoherencies that are seen in his account.

Take the problem discussed by Bratman: the need for a specific causal verb. From a forward perspective it does not make sense to exclude the generic verb to cause from the accordion effect. But from a backward perspective it is more than obvious that it is not a case of the accordion effect. Feinberg looks at a sentence to see if he can use the accordion to squeeze out causality. Without a specific causal verb, there is no way to hide causality and the sentence would be an instance of an outright ascription of causality, the first of Feinberg’s ways of ascribing responsibility. In such a generic “A causes Y” case, it is not possible to extract an obscured causality, because the causality is open and obvious. Consequently, the accordion effect does not apply.

The same explanation also works for the other exception. Feinberg claims that the accordion affect cannot be applied to simple actions. From a forward point of view, which uses the accordion effect to create new sentences, the following problem arises: why should it be impossible to stretch out the accordion and incorporate the consequences of a simple action into the description? Feinberg probably would not deny this—in fact, it is implied by his description and examples of the accordion effect—yet it is not his perspective. He is concerned with dissecting the sentence in order to see if causality can be found in it. If he is looking at a simple action, it is immediately clear, that nothing is hidden here. So this second exception also does make sense in Feinberg’s concept. It has to be said, however, that Feinberg could have seen the other, more obvious side and pointed out that the accordion effect can be applied to simple actions as forward a tool to redescribe them in terms of their consequences but not to analyze them in terms of causality.

It seems to me, that this reading of Feinberg does more justice to his conception. It does require to see Feinberg as single-mindedly (if not narrow-mindedly) pursuing his goal and sticking (or being limited) to his point of view, but in exchange, one gets a coherent account of the accordion effect, which avoids the artificiality that Bratman objected to. The difference between *principle F* and *principle D* is merely one of direction. Feinberg simply played Davidson’s accordion backwards.

## Endnotes

^a^ Feinberg first published this essay in 1965. A revised version of it was published as part of a collection of his essays in 1970. This paper is discussing the later version.

^b^ Macklin (1967) has taken Feinberg to hold that the accordion effect affects the actions themselves, not merely their description, which according to Macklin, would be correct. This interpretation stuck with Feinberg. See Aguilar (2007) for the most recent wording of it. I’m unsure if Feinberg really saw things that way, since he does also speak about “ways of talking,” “describing” and “a feature of language.” In any case I agree with Macklin and Davidson that the accordion effect works on the level of descriptions. Every time I speak about incorporating consequences into actions, it is just an elliptical way of saying the actions are being redescribed in terms of their consequences.

^c^ See Ladd (1965), Oldenquist (1966), Macklin (1967), Atwell (1969).
